# *SfCYP6AN4*-Mediated Spinetoram Resistance and RNA Pesticide Application in *Spodoptera frugiperda*

**DOI:** 10.3390/insects17050460

**Published:** 2026-04-28

**Authors:** Longyu Yuan, Danfeng Yu, Jingxuan Wang, Yanfang Li, Yangshuo Dai, Hanxiang Xiao, Zhenfei Zhang

**Affiliations:** 1 Guangdong Provincial Key Laboratory of High Technology for Plant Protection, Plant Protection Research Institute, Guangdong Academy of Agricultural Sciences, Guangzhou 510640, China; yuanlongyu@gdaas.cn (L.Y.); danfengyu@stu.scau.edu.cn (D.Y.); bravory@163.com (J.W.); liyanfang@gdaas.cn (Y.L.); daiysh@gdppri.com (Y.D.); 2 College of Plant Protection, South China Agricultural University, Guangzhou 510651, China

**Keywords:** fall armyworm, *CYP6AN4*, spinetoram, RNAi, nano-pesticide

## Abstract

Globally, the invasive fall armyworm (*Spodoptera frugiperda*, FAW) has been characterized by the widespread development of tolerance to multiple insecticides, a process facilitated by extensive and prolonged exposure to these compounds. This study utilized RNA interference (RNAi) to explore the potential association of cytochrome P450 genes to spinetoram resistance in *S. frugiperda*, with the parallel aim of investigating nanocarrier-based P450-targeting formulations as synergistic agents for improved pest management. Bioassay results from Guangdong *S. frugiperda* populations revealed the highest tolerance in the Meizhou population (LC_50_ = 0.673 µg/mL). Spinetoram exposure induced a 6.5 U/mL increase in larval P450 activity and triggered marked upregulation of *CYP6AN4* (4.98-fold versus control), which exhibited concentration-dependent induction (2.52-fold under tested conditions). The LDH-ds*CYP6AN4* + spinetoram formulation achieved 62.87% mortality, representing an 11.31% enhancement over the LDH + spinetoram group, and maintained robust synergy across diverse field populations. These phenotypic and molecular observations indicate that *CYP6AN4* upregulation is closely correlated with the spinetoram response and may potentially contribute to altered insecticide susceptibility in field populations.

## Simple Summary

Insecticide resistance is mainly mediated by multiple genes encoding diverse detoxification enzymes, among which cytochrome P450 monooxygenases (CYPs) play a crucial role in metabolizing synthetic insecticides and other toxic compounds in insects. This study investigated spinetoram resistance in the fall armyworm (*Spodoptera frugiperda*), with a focus on characterizing the function of the *CYP6AN4* gene. Following spinetoram exposure, an overall elevation in P450 enzyme activity was detected. Analysis of multiple P450 genes showed that *CYP6AN4* was significantly upregulated. Consequently, a combined treatment of layered double hydroxides (LDHs)-dsRNA and spinetoram was applied, which synergistically induced a significant increase in larval mortality.

## 1. Introduction

The fall armyworm (FAW), *Spodoptera frugiperda* (Lepidoptera: Noctuidae), is native to the tropical and subtropical Americas. Its strong migratory ability has enabled its global spread, establishing it as a major invasive pest and a significant threat to agriculture worldwide [1]. *S. frugiperda* is recognized as a polyphagous pest responsible for significant economic damage. Its major host plants comprise maize, sorghum, rice, wheat, and sugarcane [2].

To date, chemical control remains the dominant strategy for managing *S. frugiperda* [3];the key insecticides employed in this approach include spinetoram, chlorantraniliprole, dinotefuran, and related compounds [4]. However, the intrinsic biological traits of *S. frugiperda*, such as high genetic plasticity and fecundity, interact with the sustained selection pressure from frequent insecticide applications to accelerate the evolution of resistance in this pest [5]. Moreover, the improper application of insecticides can also result in varying degrees of resistance to compounds such as chlorpyrifos, chlorantraniliprole, deltamethrin, and flubendiamide [6]. To elucidate the mechanisms of resistance in *S. frugiperda* necessitates investigating its physiological and biochemical alterations under insecticide stress. As a generalized stress response, exposure to insecticides triggers a cascade of defense mechanisms to counteract toxicity—a pattern documented in diverse insect taxa such as *Musca domestica*, *Heliothis virescens*, and *Drosophila melanogaster* [7,8,9].

For *S. frugiperda* in particular, understanding the development of insecticide resistance necessitates first investigating alterations in its detoxification enzyme systems [10]. The primary detoxification enzymes in insects include cytochrome P450 monooxygenases (P450s), glutathione S-transferases (GSTs), and carboxylesterases (CarEs), which collectively play key roles in insecticide metabolism [11]. Among these detoxification enzymes, cytochrome P450 monooxygenases (P450s) have been highlighted in recent research for their pivotal role in mediating insecticide resistance. The P450 enzyme system is a critically important oxidase system, with cytochrome P450 (P450) and cytochrome P450 reductase (CPR) as its core components. Cytochrome b5 and NADH-cytochrome b5 reductase may also be involved as auxiliary electron transfer proteins in certain P450-catalyzed reactions [12]. Recent studies have confirmed that P450 enzymes also possess non-detoxification functions: they indirectly participate in the development of insecticide resistance by regulating fatty acid metabolism (e.g., fatty acid hydroxylation, oxidation, and chain elongation) and the homeostasis of bioactive molecules derived therefrom (e.g., prostaglandins, fatty acid amides, and lipoxins) [13,14,15]. In the fall armyworm, P450 genes from the *CYP6B*, *CYP321A*, and *CYP9A* clans mediate xenobiotic detoxification in response to phytochemicals. Transcriptome profiling has further identified insecticide resistance-associated P450 genes (e.g., *CYP339A1*, *CYP6AE44*, *CYP4G75*) [16], whose upregulation upon insecticide exposure directly drives the onset and enhancement of *S. frugiperda* resistance [10]. This trend is supported by recent studies: exposure of *S. frugiperda* larvae to chlorantraniliprole, emamectin benzoate, or Bacillus thuringiensis toxins triggers distinct P450 gene upregulation, where the pronounced transcriptional enhancement of genes such as *CYP4G75*, *CYP6AB12*, and *CYP321A7* contributes to insecticide detoxification. RNAi mediates precise gene silencing through sequence-specific degradation of target mRNA by exogenous double-stranded RNA (dsRNA) [17]. As a versatile tool in functional genomics and pest management [18], the high specificity of RNAi allows for targeted gene knockdown with minimal off-target effects, supporting its utility in functional studies and precision pest management [19]. In a representative study, RNAi targeting *CYP6N23*, along with miR-285L mimic administration in Culex pipiens, caused substantial downregulation of *CYP6N23*, potentiating the efficacy of deltamethrin [20].

The effectiveness of RNAi is predominantly governed by the delivery efficiency of dsRNA to its target sites. To overcome this fundamental constraint, contemporary research is progressively focusing on advanced delivery platforms, including nanotechnology and symbiont-mediated systems. Nanoparticles, defined as particulate matter with dimensions between 1 and 100 nm, serve as versatile nanocarriers. They function to shield encapsulated active ingredients (such as dsRNA and insecticides) from environmental degradation, thereby significantly enhancing their stability under operational conditions. Additionally, certain nanoparticles can compromise the structural integrity of the insect cuticle, facilitating cuticular penetration and thus augmenting the bioavailability of the delivered agents. This dual capability substantially increases their utility as advanced delivery vehicles in pest management [21]. For example, MOM@CeO_2_ has been found to reduce reactive oxygen species levels in the brown planthopper (Nilaparvata lugens), which in turn leads to decreased activity of detoxification enzymes and downregulated expression of P450 genes [22]. Advances in nanomaterial synthesis have broadened their potential for pest control. Notably, carbon quantum dot-modified fluorescent mesoporous silica nanoparticles (FL-SiO_2_NPs; 180 nm in size) and indoxacarb-loaded nanoparticles (IN@FL-SiO_2_NPs) with a drug loading capacity of 24% have been successfully developed [23]. Beyond nanocarriers, other functional nanomaterials also show potential in regulating insect detoxification systems. Star polycation (SPc) has been established as an effective vector for delivering dsRNA in insect pest control applications.) Using SPc to deliver dsRNA into insect cells can interfere with key developmental genes, thereby disrupting normal growth and development. This method has been demonstrated to downregulate target gene expression in the black cutworm (*Agrotis ipsilon*) by approximately 60% and significantly inhibit larval growth [24]. Similarly, SPc-mediated delivery of *CYP15C1* has been demonstrated to increase larval mortality in the rice stem borer, *Chilo suppressalis* [25]. A variety of nanomaterials have been explored as promising delivery systems for dsRNA in pest management, including chitosan, liposomes, star polycations, carbon quantum dots, layered double hydroxides, guanylated polymers, branched amphiphilic peptide capsules, and cell-penetrating peptides [26]. These nanocarriers can protect dsRNA from degradation and enhance its cellular uptake, thereby improving RNAi efficiency in insects. Recent advances in these nanomaterial-based delivery strategies have been comprehensively reviewed elsewhere.

Layered double hydroxides (LDHs) are a class of anionic clay nanomaterials with a unique layered structure, high biocompatibility, and excellent loading capacity for negatively charged biomolecules such as dsRNA [27]. The positively charged LDH nanosheets can efficiently bind and encapsulate dsRNA through electrostatic interactions, effectively protecting it from nuclease degradation in the insect body and improving its cellular uptake efficiency [28]. Moreover, LDHs exhibit low toxicity to non-target organisms and environmental friendliness, making them an ideal nanocarrier for dsRNA delivery in RNAi-based pest control strategies [29]. As a representative lepidopteran model insect, *Bombyx mori* has been used to establish the first oral RNAi system combined with nanoparticles, which demonstrated efficient gene silencing and provided a key technical reference for the development of RNAi-based pest control strategies in lepidopteran pests [30].

This study was conducted to elucidate the molecular mechanism of spinetoram resistance conferred by a specific cytochrome P450 gene in *S. frugiperda.* In parallel, layered double hydroxides (LDHs) were employed as a delivery vector to co-encapsulate double-stranded RNA (dsRNA) and insecticide, enabling a dual-efficacy strategy. It will significantly enhance control efficacy against *S. frugiperda* while reducing the required insecticide dose. This operationally simple and highly effective approach establishes a novel RNA interference (RNAi)-based strategy for field management of *S. frugiperda* and provides practical insights for optimizing pesticide application.

## 2. Materials and Methods

### 2.1. Insects

Field populations of *S. frugiperda* were sampled from commercial maize fields in Boluo, Zhuhai, Shenzhen, and Nanning, China, respectively. All field populations were collected by the Plant Protection Research Institute, Guangdong Academy of Agricultural Sciences. Specifically, the Nanning population was sampled in September 2024 from maize plants grown in the experimental fields of the Plant Protection Research Institute, Guangxi Academy of Agricultural Sciences (Nanning, Guangxi Zhuang Autonomous Region, China), where no insecticides had been applied for at least one month prior to sampling. Field-collected larvae were transferred to controlled laboratory conditions and maintained in artificial climate chambers (Model RXZ, Ningbo Jiangnan Instrument Factory, Ningbo, China). A laboratory-susceptible strain of *S. frugiperda* (reared indoors for multiple consecutive generations) was used as the control group for susceptibility comparisons. All *S. frugiperda* populations (both field-collected and laboratory-reared) were maintained under uniform rearing parameters: a constant temperature of 27 ± 1 °C, relative humidity of 75 ± 5%, and a photoperiod regime of 16 h light: 8 h dark (L:D).

### 2.2. Relationship Between FAW Mortality and Concentration

A leaf-dipping bioassay was performed to evaluate the toxicity of spinetoram against a laboratory-maintained susceptible strain of *S. frugiperda*, employing early second to third instar larvae as test subjects. A stock solution of spinetoram was prepared at an initial concentration of 1.0 µg/mL, followed by a two-fold serial dilution to produce a gradient of seven concentrations, with the lowest concentration reaching 0.0156 µg/mL. These dilutions were used as treatment media for the bioassay. Aliquots of artificial diet slices were fully submerged in the respective spinetoram solutions, air-dried under ambient conditions, and subsequently provided to the test larvae. Diet slices treated with double-distilled water (ddH_2_O) served as the control group. Each treatment was performed with three independent biological replicates, and three technical replicates were set for each biological replicate, with 15 s-instar larvae allocated to each replicate. Following a 48 h exposure period, surviving larvae (≤3 individuals per rearing tube) were harvested and immediately stored at −80 °C for subsequent RNA extraction.

The laboratory-susceptible strain was used as the susceptible benchmark for resistance assessment. LC_50_ values and their 95% confidence intervals (CI) were determined for each population. Resistance ratios (RR) were calculated as the LC_50_ of each field population relative to that of the susceptible strain.

### 2.3. Total RNA Extraction and cDNA Synthesis

Total RNA was extracted from *S. frugiperda* larvae using the TransZol Up Plus RNA Kit (TransScript^®^, Beijing, China), following a Trizol-based method. The obtained RNA was stored at −80 °C. Reverse transcription was performed using the One-Step gDNA Removal and cDNA Synthesis SuperMix (TransScript^®^, Beijing, China) according to the manufacturer’s instructions. The synthesized complementary DNA (cDNA) was preserved at −20 °C for subsequent gene amplification and expression analysis. For genomic DNA extraction, a DNA gel extraction kit (Axygen, Union City, CA, USA) was employed. PCR amplification of target gene fragments was conducted using either high-fidelity PCR enzyme or 2× Taq enzyme (Vazyme, Nanjing, China), with Biorun Magic PCR Mix (Biorun, Guangzhou, China) utilized for conventional PCR setups. Quantitative real-time PCR (qPCR) was performed using the 2× RealStar Green Fast Mixture (GenStar, Beijing, China). The reference gene used for qPCR normalization was validated for stability under our experimental conditions. Standard curves were constructed to determine qPCR amplification efficiency, and only primer sets with efficiency between 90% and 110% were used for subsequent analysis.

### 2.4. dsRNA Preparation and Post-RNAi Gene Expression

Primers for dsRNA synthesis (dsCYP6AN4-F: 5′-TAATATACGGAACTCACTATATAGGGTCCATCAGCGGAGAATGACGAGC-3′; dsCYP6AN4-R: 5′-TAATATACGGAACTCACTATATAGGGTGGGCAGGTA CGTCGAA CTGAAT-3′) were designed with T7 promoter sequences at the 5′ end. dsRNA was synthesized using the T7 RiboMAX™ Express RNAi System (P1700, Promega Corporation, Madison, WI, USA) according to the manufacturer’s instructions. The synthesized dsRNA (ds*CYP6AN4*) was diluted to a working concentration of 2000 ng/µL. In each Petri dish, precisely cut diet discs and ten second-instar *S. frugiperda* larvae were placed. Each dish received five sprays from a low-volume spray bottle containing the dsRNA solution. Each treatment included three independent biological replicates, with three technical replicates per biological replicate. Sampling commenced three days post-treatment, during which three live larvae from each replicate were collected into the same centrifuge tube. Larvae treated with ddH_2_O spray served as the control group. Changes in gene expression were subsequently validated by quantitative real-time PCR (qRT-PCR). All qRT-PCR analyses were performed with three biological replicates and three technical replicates per biological replicate.

### 2.5. Mortality of FAW After RNAi Treatment

Following dilution of dsRNA (ds*CYP6AN4*) to 2000 ng/µL, precisely cut diet pieces along with ten second-instar *S. frugiperda* larvae were placed in each petri dish. Each dish received five sprays from a low-volume spray bottle containing the dsRNA solution. Mortality rates were recorded after 72 h of exposure. Each treatment was performed with three biological replicates and three technical replicates per biological replicate.

### 2.6. Preparation of Nano-Pesticides

A 0.01% (*w*/*v*) magnesium-aluminum layered double hydroxide (MgAl-LDH) suspension was synthesized in the laboratory. MgAl-LDH is a classic layered nanocarrier with positively charged laminar structure and exchangeable interlayer anions, which is suitable for dsRNA loading and delivery. This suspension was then combined with *CYP6AN4*-targeting double-stranded RNA (ds*CYP6AN4*) at a mass ratio of 1:10 (LDH:dsRNA), mixed thoroughly, and incubated at room temperature for 20 min to form the final nanocarrier complex, designated LDH-ds*CYP6AN4*. The LDH-dsRNA complex preparation method was based on established protocols for nucleic acid delivery in insects [26].

### 2.7. Relationship Between Nano-Pesticides and FAW Mortality

*S. frugiperda* larvae were starved for 4 h and uniformly sprayed with the prepared LDH-ds*CYP6AN4* complexes. The complexes were then mixed separately with spinetoram (0.125 µg/mL) at a 1:1 volume ratio and applied simultaneously. All treatments were performed with three biological replicates and three technical replicates. Mortality rates were assessed after 4 days of treatment.

### 2.8. Data Analysis

All data were analyzed using Student’s *t*-test and one-way analysis of variance (ANOVA). Multiple pairwise comparisons among means were conducted using the LSD post-hoc test. Differences with *p* < 0.05 were considered statistically significant. Corrected mortality was calculated using Abbott’s formula.

## 3. Result

### 3.1. Analysis of Spinetoram Resistance in the FAW

**Figure 1:**
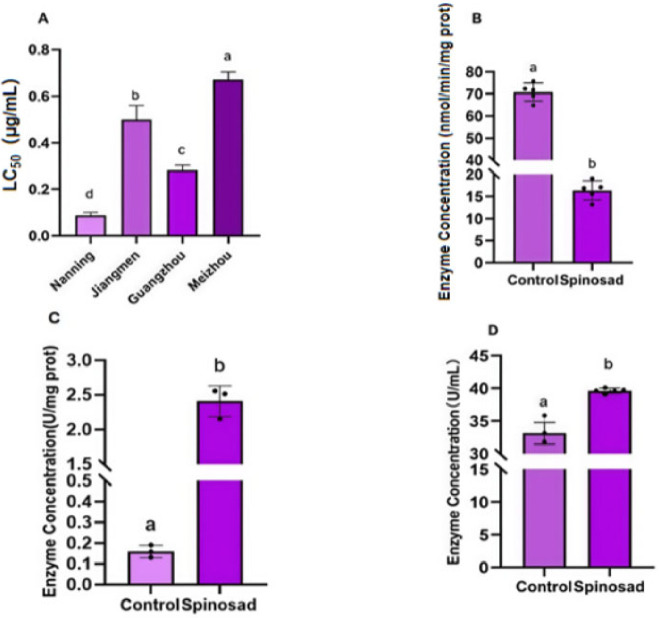
(**A**) LC_50_ values of spinetoram in different field populations of *Spodoptera frugiperda*. Effects of spinetoram exposure on the activities of glutathione S-transferase (GST, (**B**)), carboxylesterase (CarE, (**C**)), and cytochrome P450 (**D**) in *Spodoptera frugiperda*. Data are presented as mean ± standard error (SE) of three biological replicates with three technical replicates per biological replicate. Data in panel (**A**) were analyzed by one-way ANOVA followed by LSD post hoc test for multiple comparisons. Data in panels (**B**–**D**) were analyzed by Student’s *t*-test to compare significant differences between the control and spinetoram-treated groups. Different letters indicate significant differences at *p* < 0.05.

To assess spinetoram resistance in *S. frugiperda*, the resistance levels of several geographic populations from Guangdong Province were evaluated and compared. Following exposure to spinetoram, the activities of glutathione S-transferase (GST), carboxylesterase (CarE), and cytochrome P450 were measured to determine their involvement in the resistance mechanism. Bioassay results identified the Meizhou population as the most resistant, with a median lethal concentration (LC_50_) of 0.673 µg/mL, whereas the Nanning population was the most susceptible (LC_50_ = 0.089 µg/mL) (Figure 1A). Using an untreated laboratory-susceptible strain as a control, post-treatment enzyme analysis revealed that GST activity was inhibited by 77.05% (Figure 1B), and CarE activity was assayed with the untreated laboratory population of *S. frugiperda* as the control. The control group exhibited a CarE activity of 0.160 U/mg, whereas spinetoram treatment significantly increased the activity to 2.449 U/mg, with a net increase of 2.289 U/mg relative to the control. (Figure 1C), and cytochrome P450 activity increased by 6.5 U/mL to 39.62 U/mL (Figure 1D). These findings demonstrate inter-population variation in spinetoram resistance and indicate that spinetoram inhibits GST and CarE activities while elevating cytochrome P450 activity, collectively implicating these metabolic enzymes in the resistance phenotype.

### 3.2. P450 Gene-Mediated Resistance to Spinetoram

**Figure 2:**
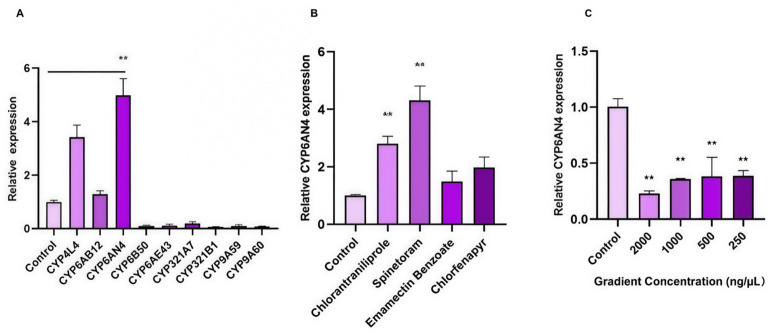
(**A**) Expression profiles of cytochrome P450 (P450) genes in *Spodoptera frugiperda* in response to spinetoram treatment; (**B**) effects of four different insecticides on *CYP6AN4* gene expression in *Spodoptera frugiperda*; (**C**) RNA interference (RNAi) efficiency of ds*CYP6AN4* at different concentrations in *Spodoptera frugiperda*. Data are presented as mean ± standard error of the mean (SEM) of three biological replicates with three technical replicates per biological replicate (*n* = 3 biological replicates). All data were analyzed by one-way ANOVA followed by the LSD post hoc test, using SPSS 26 software was deleted. Double asterisks (**) above the bars indicate highly significant differences at *p <* 0.01.

Subsequent analysis of cytochrome P450 (CYP) gene expression following spinetoram exposure focused on nine genes from the CYP4, CYP6, CYP3, and CYP9 families. *CYP6B50* and *CYP321B1* were the most downregulated (0.097- and 0.073-fold versus control, respectively), while *CYP6AN4* showed the most pronounced upregulation at 4.98-fold (Figure 2A). Due to this marked induction, we examined the insecticide-specificity of *CYP6AN4* expression by comparing its levels after exposure to four different insecticides. Significant upregulation was observed in response to spinetoram (4.31-fold) and chlorantraniliprole (2.81-fold), with more moderate induction by chlorfenapyr (1.98-fold) and emamectin benzoate (1.49-fold) (Figure 2B). To explore the potential association between *CYP6AN4* expression and spinetoram susceptibility, we evaluated the efficiency of its dsRNA-mediated silencing. A dsCYP6AN4 concentration of 2000 ng/µL achieved 77.1% interference efficiency, while 250 ng/µL reached 58.4%. Expression levels were correspondingly reduced to 0.358-, 0.382-, and 0.416-fold of the control at 1000, 500, and 250 ng/µL, respectively (Figure 2C), indicating effective knockdown of CYP6AN4 expression across tested concentrations.

### 3.3. CYP6AN4 Confers Resistance in the FAW

**Figure 3:**
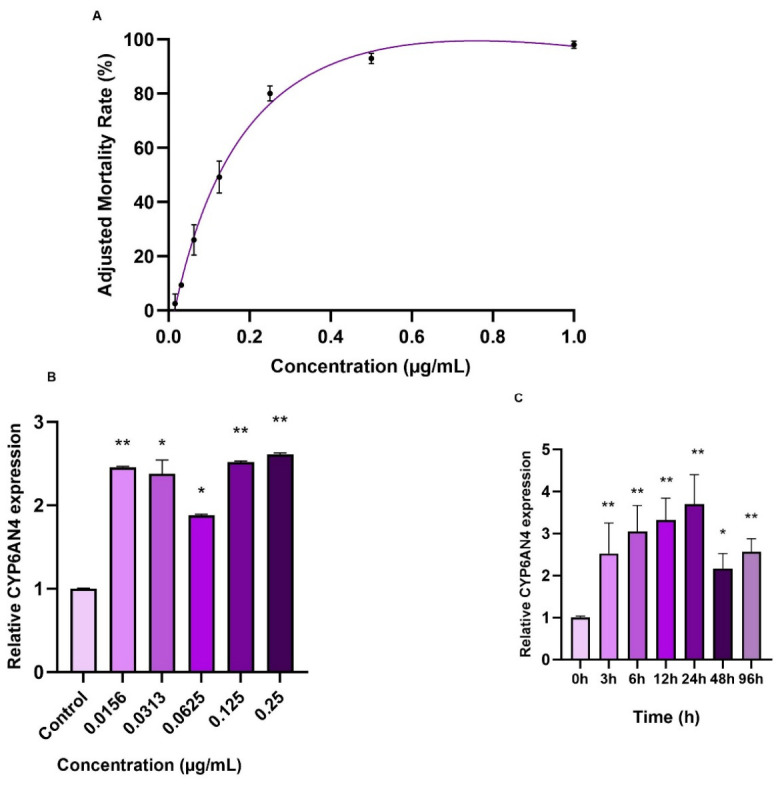
The nonlinear regression curve equation describing the relationship between spinetoram concentration and corrected mortality in the FAW is y = 26.162 ln(x) + 105.55, with R^2^ = 0.9665 (**A**). Expression levels of *CYP6AN4* in the FAW under different spinetoram concentrations (**B**) and at various time points post-treatment (**C**). Data were analyzed by one-way ANOVA (LSD method). Asterisks above the bars indicate significant differences: * *p* < 0.05, ** *p* < 0.01. Data are presented as mean ± SEM (*n* = 3).

To further characterize spinetoram resistance in *S. frugiperda*, resistance monitoring was performed, and a nonlinear regression model was established. The high R^2^ value indicated a strong fit of the model to the concentration–mortality relationship. Corrected mortality increased progressively with rising spinetoram concentration and stabilized around 0.5 µg/mL (Figure 3A). *CYP6AN4* expression was then measured in larvae exposed to different spinetoram concentrations. Overall expression showed an upward trend upon spinetoram stimulation, with the lowest level observed at 0.0625 µg/mL (1.886-fold of the control) and stabilization at 0.125 and 0.25 µg/mL (2.519-fold and 2.611-fold of the control, respectively). Significant differences were detected between the control and the 0.0156, 0.0313, 0.125, and 0.25 µg/mL treatment groups (Figure 3B). Temporal expression analysis after spinetoram exposure revealed that *CYP6AN4* peaked at 24 h (4.141-fold relative to 0 h) and, despite some fluctuation, maintained an overall upward trend compared with the control over time (Figure 3C).

### 3.4. Control Efficacy of dsRNA-Loaded Nano-Pesticides Against Field Populations of FAW

**Figure 4:**
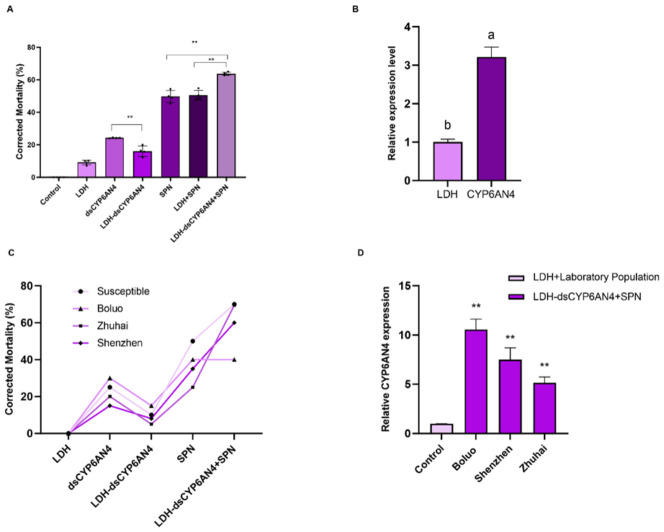
Effects of different nanopesticide treatments on the corrected mortality of *Spodoptera frugiperda* (**A**); expression of *CYP6AN4* in *Spodoptera frugiperda* following treatment with LDH-dsRNA combined with spinetoram (**B**); corrected mortality of different field populations of *Spodoptera frugiperda* after nanopesticide treatment (**C**); and *CYP6AN4* expression levels in different field populations of *Spodoptera frugiperda* after nanopesticide application (**D**). Data are presented as mean ± standard error of the mean (SEM) of three biological replicates with three technical replicates per biological replicate (*n* = 3). All data were analyzed by one-way ANOVA followed by LSD post hoc test. Different lowercase letters indicate significant differences at *p* < 0.05; asterisks above the bars indicate significant differences: *p* < 0.01 (**).

To evaluate the bioefficacy of dsRNA-based nanopesticides, we first assessed their lethal effects across different formulations. The LDH-ds*CYP6AN4* + spinetoram treatment achieved a corrected mortality of 62.87%, representing an 11.31% increase over the LDH + spinetoram group and a 13.64% increase over spinetoram alone (Figure 4A). To explore the potential association between improved insecticidal performance and target gene abundance, we examined changes in *CYP6AN4* expression following different treatments. In larvae treated with the combined formulation, the transcriptional level of *CYP6AN4* was altered to 3.21-fold of the control level (Figure 4B). To validate field applicability, mortality assays were extended to one susceptible and three field-collected populations. The Shenzhen population exhibited the highest corrected mortality (71.87%), indicating the greatest susceptibility, likely due to its lower inherent resistance (Figure 4C). Furthermore, analysis of *CYP6AN4* expression in these field populations post-treatment revealed a consistent pattern: all field populations showed higher expression than the laboratory control. The Boluo population displayed the most pronounced upregulation (11.02-fold), with statistically significant differences also observed among the field populations themselves (Figure 4D).

## 4. Discussion

The present study was performed under laboratory conditions to characterize the potential role of *CYP6AN4* in spinetoram detoxification in *S. frugiperda*. Although chemical control remains the principal approach for managing *S. frugiperda*, the intrinsic biological traits of this pest have led to varying levels of resistance to numerous insecticides [31]. This aligns with earlier reports of geographically distinct populations showing differential susceptibility to compounds such as chlorpyrifos, permethrin, and chlorantraniliprole [6]. In our study, significant inter-population variation in spinetoram resistance was observed among four geographical populations of *S. frugiperda*, with the Meizhou population exhibiting the highest resistance (LC_50_ = 0.673 µg/mL) and the Nanning population the lowest (LC_50_ = 0.089 µg/mL). Metabolic detoxification enzymes are widely recognized as key mediators of insecticide resistance in lepidopteran pests. For example, exposure to acephate, indoxacarb, beta-cypermethrin, and spinetoram has been shown to alter glutathione S-transferase (GST) gene expression in *Plutella xylostella* [32], while treatment with chlorantraniliprole at its LC_50_ significantly elevated cytochrome P450 and GST activities while suppressing carboxylesterase (CarE) activity in *S. frugiperda.* Correspondingly, our results demonstrated that spinetoram exposure differentially regulated the activities of key metabolic enzymes in *S. frugiperda*: it inhibited GST activities while inducing cytochrome P450 and CarE activity. This concerted yet opposite modulation underscores the central, yet complex, role of these enzymes in the insect’s response to insecticide stress, which may facilitate the development of higher tolerance in field populations.

A central finding of this study is the insecticide-specific modulation of detoxification enzymes in *S. frugiperda*, with cytochrome P450 identified as the principal mediator of spinetoram resistance. This response is consistent with the documented role of P450 overexpression in metabolic resistance [33], as exemplified by the association of *Plutella xylostella* CYP6BG1 with chlorantraniliprole resistance [34] and the increased insecticide susceptibility resulting from the suppression of P450 genes such as *CYP9F2* [35]. Our data provide direct evidence for this mechanism: spinetoram induced a concentration-dependent upregulation of the core P450 gene *CYP6AN4*, with expression reaching 4.98-fold that of the control. This induction suggests that spinetoram directly triggers *CYP6AN4* overexpression, which may contribute to the insecticide’s metabolic detoxification. Furthermore, corresponding enzyme activity assays revealed that P450 was the sole detoxification enzyme induced by spinetoram. These findings are consistent with the results of field population surveys of insecticide resistance in *Spodoptera frugiperda* in China [36], which have shown that differences in susceptibility among geographical populations are closely related to the expression levels of detoxification enzymes such as cytochrome P450, glutathione S-transferase, and carboxylesterase. Together, these results support the conclusion that P450 genes, particularly *CYP6AN4*, may participate in the spinetoram response and likely contribute to population tolerance, rather than confirming a definitive causal role in metabolic resistance.

RNA interference (RNAi) provides a powerful tool for investigating gene function in insects and developing targeted pest control strategies. In this study, silencing of the *CYP6AN4* gene via RNAi achieved an interference efficiency of 77.10% using 2000 ng/µL dsCYP6AN4. Bioassays confirmed that spinetoram stress induces *CYP6AN4* upregulation. When RNAi-mediated knockdown was combined with spinetoram treatment at the LC_50_, the Boluo population displayed the highest expression level (7.69-fold), which nevertheless represented a 25.1% reduction compared to its non-RNAi counterpart. These results reflect population-specific differences in response to both insecticide exposure and RNAi. However, the practical application of RNAi in pest management faces several obstacles. In lepidopterans, for instance, factors such as alkaline gut pH and dsRNA degradation by nucleases can limit RNAi efficacy [37]. Additional challenges include variable interference efficiency across species, the absence of robust delivery systems, and potential off-target or non-target effects [38]. Consequently, research efforts are increasingly directed toward novel delivery platforms designed to improve dsRNA stability and cellular uptake.

In recent years, spray-induced gene silencing (SIGS) has gained considerable attention as a novel approach to agricultural pest control, wherein topically applied nucleic acid formulations trigger silencing of target genes on plant surfaces [39]. For instance, a sprayable formulation comprising dsRNA complexed with star polycation (SPc) achieved 61% control of the green peach aphid at 3 days post-application, with efficacy remaining near 50% by day 6 [40]. LDH nanoparticles exhibit excellent colloidal stability in aqueous environments, and their particle size, zeta potential, and dispersion are closely related to environmental conditions, providing a reliable basis for the evaluation of LDH-based delivery systems [41]. In the present study, a similar strategy was adopted by formulating dsRNA targeting CYP6AN4 with layered double hydroxide (LDH) nanocarriers, followed by combination with spinetoram. This combined application significantly enhanced insecticidal efficacy, with the LDH-dsCYP6AN4 + spinetoram treatment reaching 62.87% mortality—an 11.31% increase over the LDH + spinetoram control group, thereby effectively reducing *S. frugiperda* tolerance to the insecticide. Moreover, when applied to different field-collected populations, the nanopesticide increased mortality across all tested strains, confirming its broad synergistic potential in managing wild populations of *S. frugiperda*.

RNAi efficiency may differ among field populations with varied genetic backgrounds and resistance levels, as widely reported in lepidopteran pests [37]. Although the physicochemical characterizations confirmed the favorable stability, loading capacity, and particle properties of LDH nanocarriers under laboratory conditions, their long-term environmental fate, persistence, and biosafety in realistic paddy field ecosystems still require further in-depth investigation. These issues are consistent with current challenges for nano-enabled RNAi delivery systems, and future work should focus on field-scale evaluation of stability, deposition, and non-target impacts to support the practical application of LDH-based dsRNA formulations. Further field-scale studies are needed to confirm the consistency and reliability of these findings under practical application conditions.

## 5. Conclusions

**Figure 5:**
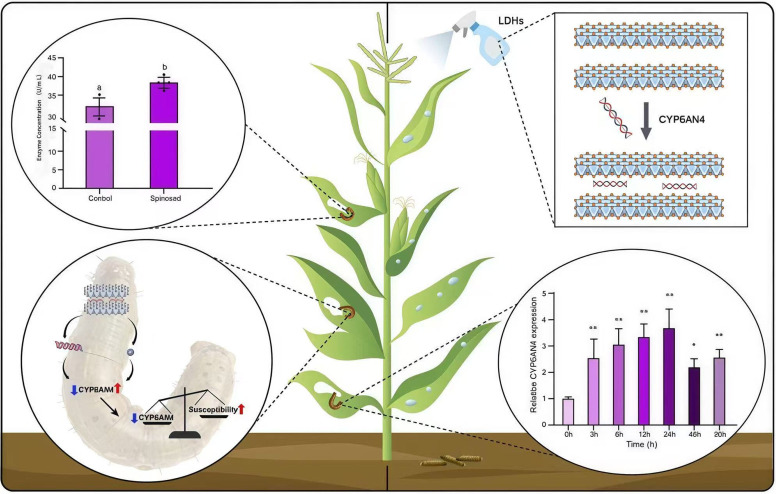
The *CYP6AN4* gene is implicated in FAW spinetoram resistance, and co-application of its targeted nano-pesticide LDH-dsCYP6AN4 with spinetoram significantly elevates mortality rate. Data are presented as mean ± SD. * *p* < 0.05, ** *p* < 0.01 vs. Control group. Different lowercase letters indicate significant differences between groups (*p* < 0.05).

This study demonstrated that spinetoram exposure significantly induces cytochrome P450 activity in *S. frugiperda*, with the key P450 gene *CYP6AN4* showing clear concentration-dependent upregulation. RNA interference assays further established that *CYP6AN4* directly contributes to spinetoram resistance, representing a central molecular mechanism in the insect’s response to insecticide stress. Building on this finding, we developed an RNAi-based nanocarrier formulation (LDH-ds*CYP6AN4*) as a pesticide synergist. This formulation significantly enhanced insecticidal efficacy, achieving 62.87% corrected mortality, and demonstrated strong synergistic activity against wild populations of *S. frugiperda* (Figure 5). In summary, our work elucidates the pivotal role of *CYP6AN4* in spinetoram resistance and provides a targeted, sustainable pest management strategy that supports the development of environmentally rational control practices while reducing dependence on conventional chemical pesticides.

## Data Availability

The original contributions presented in this study are included in the article. Further inquiries can be directed to the corresponding author.
